# Predictive and preventive significance of AMPK activation on hepatocarcinogenesis in patients with liver cirrhosis

**DOI:** 10.1038/s41419-018-0308-4

**Published:** 2018-02-15

**Authors:** Xiaoli Yang, Yan Liu, Menghui Li, Hao Wu, Yunbing Wang, Yu You, Peizhi Li, Xiong Ding, Chang’an Liu, Jianping Gong

**Affiliations:** 1 Chongqing Key Laboratory of Hepatobiliary Surgery and Department of Hepatobiliary Surgery, The Second Affiliated Hospital of Chongqing Medical University, Chongqing, 400010 People’s Republic of China; 2 Department of Gastroenterology, the Fifth People’s Hospital of Chengdu, Chengdu, Sichuan 611130 People’s Republic of China

## Abstract

Metformin has been demonstrated to prevent hepatocellular carcinoma (HCC). Metformin acts mainly by phosphorylation of AMPK. However, the phosphorylation status of AMPK and its role in the prediction and prevention of HCC in cirrhotic patients remains unclear. The phosphorylation status of AMPK (Thr172) was determined by immunostaining in tissue microarrays of 426 cirrhotic liver tissues. Low expression of p-AMPK was observed in 94 (22.1%) cases. The median follow-up time was 87 months. HCC occurrence probability at 1/3/5/10 years after Hassab procedure was 3.1/9.6/13.8/30.6% in patients with p-AMPK low expression and 0/0.3/0.3/8% in patients with p-AMPK high expression, respectively. HCC occurrence risk was significantly higher in patients with p-AMPK low expression in univariable analysis (HR, 6.25; 95% CI: 3.36–11.60; *P* < 0.001) and multivariable analysis (HR, 6.0; 95% CI: 3.24–11.10; *P* < 0.001). An independent external cohort validated the significance of p-AMPK low expression. In addition, in vivo experiments demonstrated that AMPK activation status was negatively related to HCC occurrence and blocking autophagy by chloroquine counteracted the protective effect of AMPK phosphorylation. These results present novel insight into a critical predictive role of AMPK activation in hepatocarcinogenesis and AMPK activation seems to be a potential target for the prevention of hepatocellular carcinoma in patients with liver cirrhosis.

## Introduction

Liver cirrhosis is the 13th leading cause of death globally, with worldwide mortality having increased by 45.6% from 1990 to 2013^1^. Cirrhosis is a milieu that is conducive to development of hepatocellular carcinoma (HCC), the second most lethal cancer worldwide^2^. Therefore, it is of vital importance to identify novel effective prediction and prevention strategies for the management of liver cirrhosis. Metformin is a first-line antidiabetic drug. In recent years, metformin has been suggested to play a role in the suppression of various cancers in patients with diabetes, including HCC, gastric cancer, colorectal cancer, etc^3–5^.

Metformin exerts pharmacological effects mainly through the phosphorylation of the AMP-activated protein kinase (AMPK), a highly conserved heterotrimeric serine/threonine kinase^6,7^. In response to certain stressful conditions, such as energy deprivation, AMPK is activated by phosphorylation at Thr172 in α subunit. Once activated, AMPK regulates various molecules and signaling pathways to modulate adaptive changes and maintain metabolic homeostasis^7–9^. Recently, emerging evidence has indicated the potential role of AMPK signaling in tumor initiation and progression^8,10,11^. An early finding showed that AMPK pathway activation by metformin was involved in metformin-induced growth inhibition of cancer cells^12^. Further, AMPK activation not only downregulated mTOR but also suppressed the excess aerobic glycolysis (Warburg effect) characteristic of most transformed cells and genetic ablation of the a1 catalytic subunit of AMPK accelerated Myc-induced lymphomagenesis^6,13^. This line of investigation suggested a tumor suppressor role for AMPK activation. AMPK is also a promising target for treating fibrotic diseases. AMPK activation reduced thioacetamide-induced liver fibrosis in mice and inhibited the activation of cultured hepatic stellate cells and macrophages^14^. These data suggested that AMPK activation was an important factor regulating both fibrosis and cancer. Therefore, it is intriguing to understand the phosphorylation status of AMPK in cirrhotic livers and its role in the process from cirrhosis to HCC.

In the present study, we use population study and in vivo experiments to address the role of p-AMPK in the pathogenesis of HCC.

## Results

### Baseline characteristics in the testing corhort

**Fig. 1:**
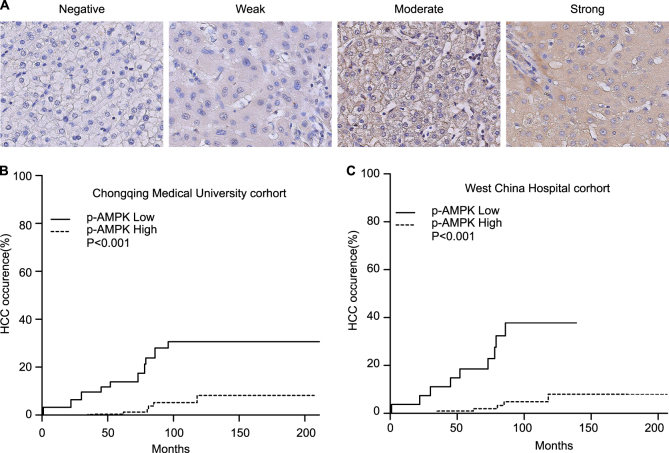
Low p-AMPK expression correlates with HCC occurrence in patients with liver cirrhosis. **a** Immunohistochemical staining of the expression of p-AMPK in cirrhotic liver tissues. **b**, **c** Cummulative probability of HCC occurrence accodring to p-AMPK expression in Chongqing and West China cohorts

On the basis of the immunohistochemical staining of p-AMPK by tissue microarrays, 426 patients with liver cirrhosis were divided into two groups: high p-AMPK expression group (331, 77.7%) and low p-AMPK expression group (94, 22.3%). Typical images were shown in Fig. 1a. As shown in Table 1, patients in low p-AMPK expression group did not differ significantly from patients in high p-AMPK expression group regarding sex, age, etiology, ALT, AST, GGT, TBIL, ALB, AFP, hepatic encephalopathy, ascite and alcohol history (*P* > 0.05). Patients in low p-AMPK expression group had a significantly higher proportion of patients with Child-pugh B or C (64.9% vs 57.2%, *P* = 0.024), diabetes mellitus (40.4% vs 29.5%, *P* = 0.045), and GI bleeding (62.8% vs 34.0%, *P* < 0.001) compared with patients in high p-AMPK expression group (Table 2). After a median follow-up of 87 months, HCC occurred in 41 (9.6%) patients and 61(14.3%) patients died.

**Table 1:** Correlation between expression of p-AMPK (Thr 172) and clinicopathological characteristics in Chongqing and West China cohort

	Chongqing cohort			West China cohort		
	p-AMPK(low)	p-AMPK(High)	*P* value	p-AMPK(low)	p-AMPK(High)	*P* value
*N*	94	332		27	104	
Gender (M)	69(73.4)	245(73.8)	0.939	18(66.7)	64(61.5)	0.624
Age	46.2 ± 10.9	46.4 ± 11.1	0.905	48.5 ± 13.8	46.3 ± 12.3	0.409
Etiology			0.066			
HBV	73(77.7)	292(88.0)		27(100)	104(100)	
HCV	3(3.1)	9(2.7)				
NAFLD	7(7.4)	14(4.2)				
Alcohol	4(4.2)	9(2.7)				
Unclear	7(7.4)	8(2.4)				
Child-pugh			0.024			0.929
A	33(35.1)	142(42.8)		13(48.1)	54(51.9)	
B	48(51.1)	171(51.5)		13(48.1)	47(45.2)	
C	13(13.8)	19(5.7)		1(3.7)	3(2.9)	
ALT(IU/L)	53.9 ± 38.6	53.6 ± 43.4	0.994	59.6 ± 33.9	41.5 ± 29.4	0.007
AST(IU/L)	67.2 ± 48.0	65.3 ± 49.2	0.741	67.519 ± 42.845	53.3 ± 37.0	0.088
GGT(IU/L)	72.3 ± 58.0	69.1 ± 59.3	0.646	81.963 ± 54.886	70.4 ± 63.5	0.39
AFP(μg/L)	34.9 ± 74.2	32.9 ± 73.4	0.811	23.584 ± 27.272	26.4 ± 48.6	0.772
TBIL(μmol/L)	42.9 ± 48.8	35.9 ± 36.5	0.318	44.311 ± 58.939	30.1 ± 27.8	0.913
ALB(g/dL)	3.7 ± 0.7	3.7 ± 0.6	0.817	3.775 ± 0.523	3.7 ± 0.6	0.731
Ascite	22(23.4)	72(21.7)	0.723	6(22.2)	21(20.2)	0.816
Hepatic encephalopathy	1(1.1)	3(0.9)	1.000	1(3.7)	0(0)	0.206
Diabetes mellitus	38(40.4)	98(29.5)	0.045	8(29.6)	31(29.8)	0.986
Alcohol	22(23.2)	76(22.9)	0.917	5(18.5)	20(19.2)	0.933
GI bleeding	59(62.8)	113(34.0)	<0.001	7(25.9)	41(39.4)	0.195

**Table 2:** Characteristics of patients in Chongqing and West China cohorts

Variable	Chongqing cohort(426)	West China cohort (131)	*P*
Gender(M)	314(73.7)	82(62.6)	0.014
Age	46.4 ± 11.1	46.7 ± 12.6	0.247
Etiology			<0.001
HBV	365(85.7)	131(100)	
HCV	12(2.8)		
NAFLD	21(4.9)		
Alcohol	13(3.1)		
Unclear	15(3.5)		
Child-pugh			0.049
A	175(41.1)	67(51.1)	
B	219(51.4)	60(45.8)	
C	32(7.5)	4(3.1)	
ALT(IU/L)	53.8 ± 42.4	45.2 ± 31.2	0.033
AST(IU/L)	65.7 ± 48.9	56.3 ± 38.5	0.052
GGT(IU/L)	69.8 ± 59.0	72.8 ± 61.8	0.617
AFP(μg/L)	33.3 ± 73.5	25.8 ± 45.0	0.27
TBIL(μmol/L)	37.4 ± 39.6	33.0 ± 36.6	0.256
ALB(g/dL)	3.7 ± 0.7	3.7 ± 0.6	0.556
Ascite	94(22.1)	27(20.6)	0.724
Hepatic encephalopathy	4(0.9)	1(0.8)	0.852
Diabetes mellitus	136(31.9)	39(29.8)	0.642
Alcohol	98(23.0)	25(19.1)	0.344
GI bleeding	172(40.4)	48(36.6)	0.444
P-AMPK(Low)	94(22.1)	27(20.6)	0.724

### Low p-AMPK expression correlated with HCC occurrence

**Fig. 2:**
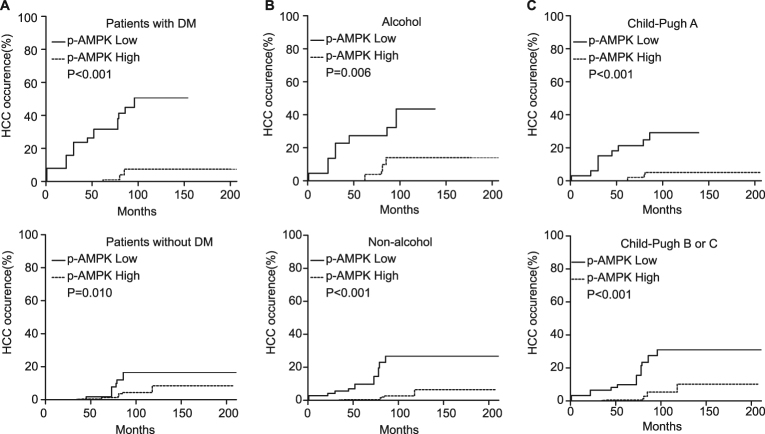
Cumulative probability of HCC occurrence according to p-AMPK expression in the subgroup of patients. **a** Patients with or without DM. **b** Patients with or without alcohol history. **c** Patients in Child-pugh A or B/C

HCC occurrence probability at 1/3/5/10 years after Hassab procedure was 3.1/9.6/13.8/30.6% in patients with p-AMPK low-expression and 0/0.3/0.3/8% in patients with p-AMPK high-expression, respectively (Fig. 1b). Competing risk analysis revealed that low p-AMPK expression was associated with HCC occurrence (Fig. 1b). In univariate analysis, low p-AMPK expression was risk factor for HCC occurrence (HR, 6.25; 95% CI: 3.36–11.60; *P *< 0.001). As shown in Table 3, low p-AMPK expression was an independent risk factor for HCC occurrence identified by multivariate analysis (HR, 6.00; 95% CI: 3.24–11.10; *P *< 0.001). Because diabetes and alcohol are known risk factors for HCC and Child-pugh grade indicates different cirrhosis degree, stratified analysis was conducted according to diabetes mellitus, alcohol, and Child-pugh grade. The results showed there was a consistent trend toward higher HCC occurrence risk in low p-AMPK expression group irrespective of DM, alcohol history or Child-pugh grade (Fig. 2a–c).

**Table 3:** Multivariate analysis of risk factors for HCC occurrence

Variables	HR	95% CI	*P* value
Chongqing cohort (*N* = 426, 94 p-AMPK low expression and 332 p-AMPK high expression)			
P-AMPK expression (low/high)	6.000	3.24–11.10	<0.001
Diabetes mellitus(Yes/no)	2.380	1.29–4.37	0.005
Alcohol(Yes/no)	2.110	1.11–3.99	0.022
West China cohort (*N* = 131, 27 p-AMPK low expression and 104 p-AMPK high expression)			
P-AMPK expression (low/high)	13.400	5.14–34.9	<0.001
Alcohol(Yes/no)	12.100	4.55–31.9	<0.001

### An independent cohort of West China Hospital confirmed the significance

The predictive value of p-AMPK expression was also evaluated in an independent cohort of 131 HBV-related cirrhosis patients from West China Hospital of Sichuan University. The median follow-up time was 84.5 months. As shown in Table 2, There was no significantly difference regarding low p-AMPK expression between West China Hospital cohort and Chongqing cohort (22.1% vs 20.6%, *P *= 0.724). Patients in West China hospital cohort did not differ significantly from patients in Chongqing cohort regarding age, AST, GGT, TBIL, ALB, AFP, ascite, hepatic encephalopathy, DM, alcohol history, and GI bleeding (*P *> 0.05). Patients in West China cohort had a significantly lower ALT and lower proportion of male and Child-pugh B or C patients. In addition, patients in West China cohort were all HBV-related. As shown in Table 1, patients in low p-AMPK expression group did not differ significantly from patients with high p-AMPK expression except a higher ALT level in West China cohort. In accordance with Chongqing cohort, competing risk analysis revealed that low p-AMPK expression was associated with HCC occurrence (Fig. 1c). Results of univariable analysis indicated that HCC occurrence risk was significantly higher in low p-AMPK expression group (HR, 8.49; 95% CI: 2.93–24.6; *P* < 0.001). As shown in Table 3, multivariable analysis confirmed the significantly higher HCC occurrence risk in low p-AMPK expression group (HR, 13.4; 95% CI: 5.14–34.9; *P* < 0.001).

### AMPK activation negatively correlated with hepatic carcinogenesis in in vivo experiment

**Fig. 3:**
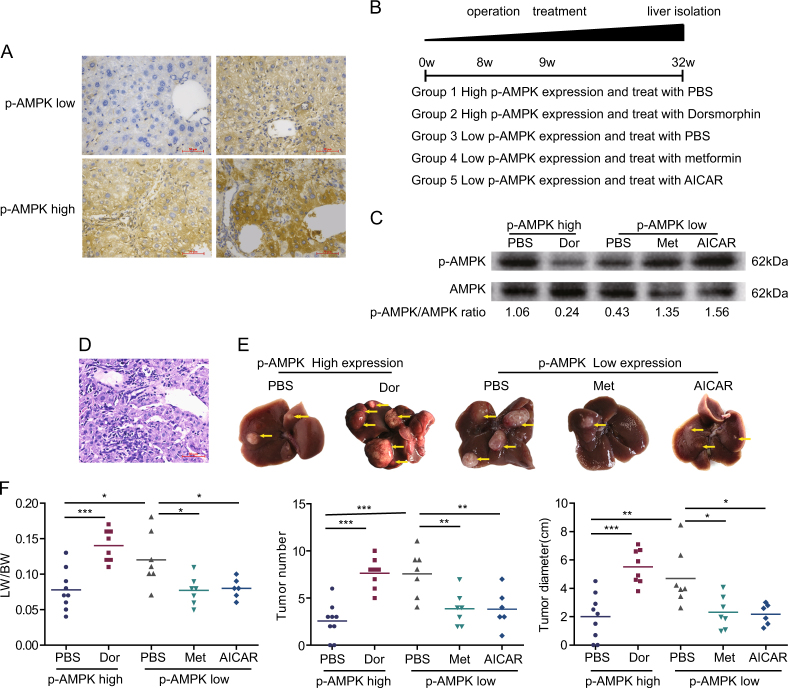
AMPK activation inhibits hepatic carcinogenesis. **a** Immunohistochemical staining of the expression of p-AMPK in mice model at 8 weeks. **b** Experimental flow chart. **c** Liver lysates from mice were analyzed by immunoblot with the indicated antibodies. **d** Hepatic carcinogenesis was confirmed by H.E. staining. **e** Tumor bearing livers 32 weeks after DEN injection; arrows indicate tumors. **f** Comparison of liver index, total tumor number and tumor diameter. Horizontal lines indicate mean values. ***P < 0.001, **P < 0.01, *P ≤ 0.05

To verify if AMPK activation can reduce HCC occurrence, we performed liver biopsy in mice at 8 weeks in the model process. The expression of p-AMPK was detected by immunohistochemical staining. The same grouping criteria with human liver samples were adopted. The typical images were shown in Fig. 3a. According to the expression of p-AMPK, 17 mice were p-AMPK high-expression and 20 mice were p-AMPK low expression. In mice with p-AMPK high expression, dorsomorphin (*n* = 8) or PBS (*n* = 9) were used to inhibit AMPK activation or as control. In p-AMPK low-expression group, metformin (*n* = 7) or AICAR (*n* = 6) were used to activate AMPK, PBS (*n* = 7) was used as control. The experimental flow chart was shown in Fig. 3b. AMPK was successfully inhibited or activated indicated by p-AMPK/AMPK ratio (Fig. 3c, Supplementary Fig. 1A). HCC occurrence was confirmed by pathology (Fig. 3d). When treated with PBS, mice with low p-AMPK expression showed higher liver index, visible tumor number, and total tumor diameter (Fig. 3e, f). Futher more, in p-AMPK high-expression mice, AMPK inhibition by dorsmorphin lead to a significant increase in liver index, visible tumor number and total tumor diameter (Fig. 3e, f). By contrast, in p-AMPK low-expression mice, a significant decrease in tumor number and total tumor diameter was observed in the metformin or AICAR treated mice compared to PBS (Fig. 3e, f). Consistent with clinical observation, the in vivo experiment implied the important role of p-AMPK activation in the process of HCC initiation. To detect potential toxicity of the activator or inhibitor, we prepared tissue sections from lung, heart, kidney, and brain of the mice that were treated with different agents. The tissue sections were analyzed by H.E. staining. The results revealed that there was no obvious abnormality in tissue anatomy structure, and no damage lesion/necrosis was observed (Supplementary Fig. 1B).

### AMPK activation improved liver inflammation and fibrosis

**Fig. 4:**
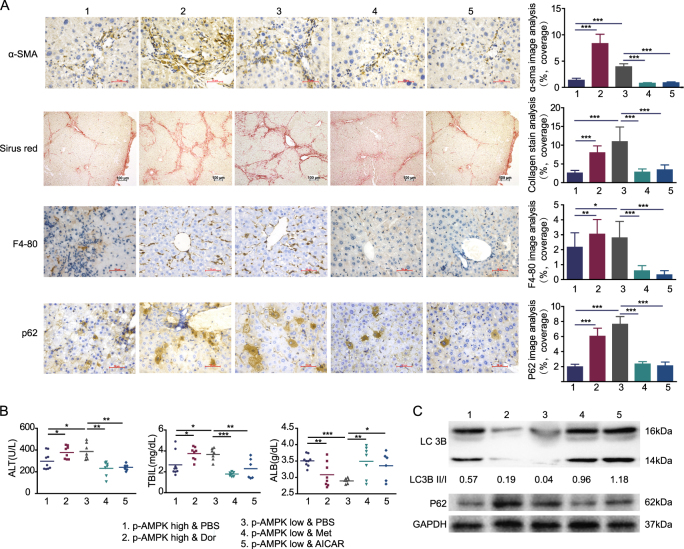
AMPK activation improve liver inflammation and fibrosis. **a** Representative images of liver section from mice with different groups that are stained with immunostained with the indicated antibodies. Graphs depict quantification of indicated parameters (mean ± SD, *n* = 6–9 mice per group). **b** Liver lysates from mice were analyzed by immunoblot with the indicated antibodies. GAPDH was used as loading control. **c** Graph depicting serum AST, TBIL, and ALB with indicated groups. Horizontal lines indicate mean values. The LC3B II/I ratios were quantitated using Image J software. ***P < 0.001, **P < 0.01, *P ≤ 0.05

Because inflammation and fibrosis are the main aspects that lead to HCC, we check if AMPK activation can relieve liver inflammation and fibrosis. Strikingly, HSC activation, fibrosis and myeloid cell were drastically increased when AMPK inhibited by dorsmorphin in p-AMPK high expression mice. Also, AMPK activation by either metformin or AICAR resulted in reduced HSC activation, fibrosis and myeloid cells (Fig. 4a). In addition, hepatic serologic injury indicators were negatively related to p-AMPK activation (Fig. 4b). It was reported that diminished hepatic lipogenesis or hepatic progenitor cell activation was involved in the protective process of metformin on hepatocarcinogenesis^4,15^. The protein levels of ACC, the rate limiting enzyme involved in fatty acid synthesis, or serum triglycerides levels were not different significantly in neither different p-AMPK expression level mice treated with PBS nor different p-AMPK modulators (Supplementary Fig. 2A-2C). We also did not observe any significant differences in the expression of RAGE, DLK-1 and CD44 (marker of hepatic progenitor cell activation) between the different groups (supplementary Fig. 2A and 2D). Taken together, our results are different from those reported in non-cirrhotic or cirrhotic livers^4,15^, and suggested that mechanisms other than diminished hepatic lipogenesis or hepatic progenitor cell activation were also responsible for the decreased incidence of HCC. Because p62 is necessary and sufficient for HCC induction in mice and the main downstream protein of AMPK-mTOR-autophagy axis^16^, we detected mTOR, LC3B, and p62 expression in each group. Our results demonstrated that AMPK activation significantly increased mTOR phosphorylation, LC3B II/I ratio and decreased p62 expression when analyzed by Western blotting (Fig. 4c, Supplementary Fig. 2A, 2E and 3A). Correspondingly, analysis of autophagy using transmission electron microscope (TEM) showed increased numbers of autophagosomes in the liver of high AMPK activation group (Supplementary Fig. 3B and 3C).

### Blocking autophagy counteracted the protective effect of AMPK phosphorylation

**Fig. 5:**
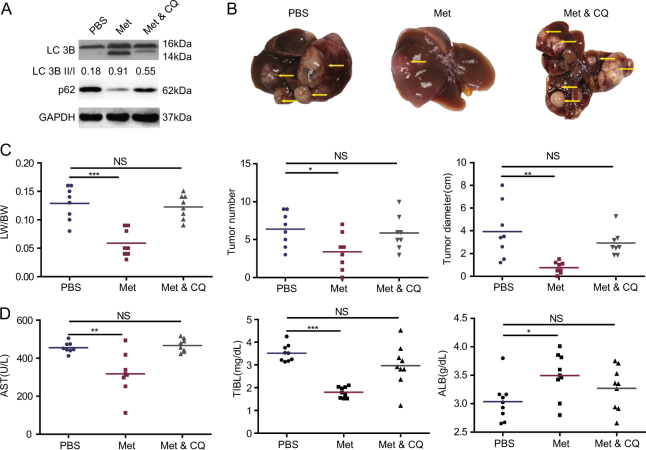
CQ counteracted the protective effect of AMPK phosphorylation. **a** Liver lysates from mice were analyzed by immunoblot with the indicated antibodies. GAPDH was used as loading control. **b** Tumor bearing livers 32 weeks after DEN injection; arrows indicate tumors. **c** Comparison of liver index, total tumor number, and tumor diameter. Horizontal lines indicate mean values. **d** Graph depicting serum AST, TBIL, and ALB with indicated groups. Horizontal lines indicate mean values. ***P < 0.001, **P < 0.01, *P ≤ 0.05, NS *P* > 0.05

**Fig. 6:**
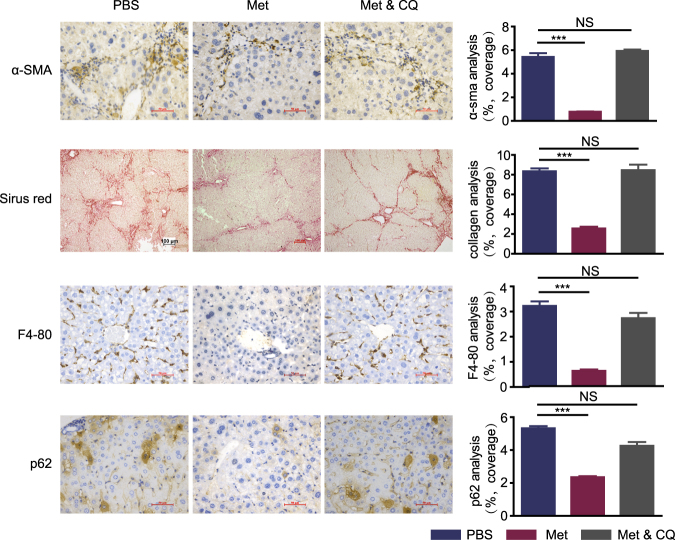
Chloroquine counteracted the protective effect of AMPK phosphorylation. Representative images of liver section from mice with different groups that are stained with immunostained with the indicated antibodies (mean ± SD, *n* = 8 mice per group). ***P < 0.001, **P < 0.01, *P ≤ 0.05, NS *P* > 0.05

To explore if autophagy plays a key role in p-AMPK-mediated protective action for HCC occurrence, we used CQ to inhibit autophagy during metformin use in p-AMPK low-expression mice (*n* = 8 mice per group). As expected, CQ decreased numbers of autophagosomes in the liver compared with metformin alone and promoted accumulation of p62 without suppressed LC3B-II production (Fig. 5a, Supplementary Fig. 4A-4C). A significant increase in tumor number and tumor diameter was observed in metformin plus CQ group than metformin alone group (Fig. 5b–d). However, there was no significant difference between the metformin plus CQ group and the control group (Fig. 5b–d). Notably, CQ removed the metformin-mediated protective action against hepatic carcinogenesis. The HSC activation, liver fibrosis and myeloid cell reduction by metformin were also blocked by CQ (Fig. 6). These data indicated that metformin inhibited hepatic carcinogenesis dependent or at least partly dependent on autophagy induction in vivo. Taken together, these data suggested that AMPK activation inhibits hepatic carcinogenesis in vivo, and the mechanism may be related to the induction of autophagy.

## Discussion

To detect the role of AMPK activation in the development of HCC, we collected two-independent cohorts with 557 cirrhotic liver tissues with a long-term follow-up. Our study, for the first time, defines a tumor suppressor role of p-AMPK in HCC occurrence based on clinical data. Furthermore, the in vivo experiment showed that p-AMPK level negatively related with hepatocarcinogenesis. In addition, we observed that autophagy induction was the downstream process involved in p-AMPK-mediated protection against hepatic carcinogenesis.

There are several caveats to these data. First, HBV related cirrhosis occupied up to 85.6% in the testing cohort and 100% in the West China cohort and all patients included in this study were certainly need of preventing or treating upper gastrointestinal bleeding by surgery. Whether p-AMPK can be applied to patients caused by other etiology or with lighter cirrhosis will need further study. Second, for the in vivo experiment was based on activator or inhibitor to modulate AMPK activation, bias resulted by the side effect of drugs may be possible. Suitable genetic mouse model are required to further validate the finds. Third, the exact mechanism involved is still to be investigated.

Several studies concentrating on the biomarker of prognosis aspects of HCC have been conducted; however, there has been little research focused on HCC occurrence. There are primarily two reasons for this; one is the difficulty of collection of cirrhotic liver tissues, especially a large number of clinical samples, and the other is the need for long term follow-up to observe HCC occurrence. In this study, we collected 557 cirrhotic liver tissues and followed up for a long enough time. This is the largest cohort examining the molecular process from liver cirrhosis to HCC in clinical specimens.

It is historically recognized that metformin is independently associated with a reduced incidence of HCC irrelevant to cirrhosis etiology in patients with cirrhosis complicated with type 2 diabetes^3,17^. Moreover, the data collected by Higurashi et al.^18^ indicated that even in patients without diabetes, the administration of low-dose metformin was safe, and metformin had a potential role in the chemoprevention of colorectal cancer. These data suggested that the role of metformin in tumor prevention may be unrelated to diabetes status. DePeralta et al.^4^ reported that metformin could prevent HCC in rat model of cirrhosis induced by DEN alone. But whether metformin exert HCC prevention effects through AMPK dependent or independent pathway is still unsolved. In the present study, we observed that AMPK activation was associated with reduced HCC occurrence in liver cirrhotic patients from population study. Futher more, p-AMPK expression is also negatively related with HCC occurrence in DEN induced mouse model and whether activating AMPK in low p-AMPK expression mice or inhibiting AMPK in p-AMPK high-expression mice could significantly change the occurrence of HCC. These data suggests that AMPK activation could be a promising biomarker and preventive target for HCC occurrence in patients with cirrhosis. On the other hand, it supports that AMPK activation is the main downstream target mediated the anticancer effect of metformin.

Autophagy, an AMPK downstream process, has well-established tumor-suppressive properties^19^. p62, which is upregulated in autophagy-deficient livers, is necessary and sufficient for HCC induction in mice, and its high expression in non-tumor human livers predicts rapid HCC recurrence after curative ablation^16^. In this study, autophagosome number and LC3 II/I increase and p62 reduction was observed after AMPK activation. In addition, autophagy disruption by CQ completely blocked the protective effect of AMPK activation. These results indicate that AMPK activation reduces HCC risk may through at least partially through autophagy induction.

In summary, our results revealed that activation of AMPK negatively correlated with HCC occurrence in clinical data and in vivo. The underlying mechanisms were autophagy-related. AMPK activation should be an attractive target for HCC prediction and prevention and provide further evidence that metformin might be considered as a potential drug for HCC prevention.

## Material and method

### Patients and tissue microarrays

We screened the records of 946 patients who underwent Hassab procedure and liver biopsy for treating liver cirrhosis between January 1999 and June 2011 in the Second Affiliated Hospital of Chongqing Medical University (Chongqing, China). In total 215 patients lacked integrated clinic data were excluded. Approximately 147 patients were excluded with insufficient tissue samples for investigation. Another 158 patients who were not following up regularly or without follow-up data were excluded. All human sample collection procedures were approved by the Ethical Review Committee of the Hospital. Informed consent was obtained in all cases before surgery. The diagnosis of liver cirrhosis was confirmed by pathology.

All patients were followed up until HCC detection or the end of the study in December 2016, with a median follow-up time of 87 months. The patients were monitored by measurement of serum alpha fetoprotein level and abdominal ultrasound every 3–6 months. When HCC was suspected, enhanced CT or MRI scanning was used to confirm the HCC diagnosis. The time from surgery to the first detection of HCC was recorded. The follow-up data were obtained through medical record system if the patients follow up in our hospital regularly. If the patients did not follow up in our hospital, the time interval of follow-up and the examination items were first checked. Only the patients followed up with the stated time interval and examination items were included in this study.

Type 2 Diabetes was diagnosed as a fasting plasma glucose level of >7.0 mmol/L (126 mg/ dL), or a plasma glucose level of >11.1 mmol/L (200 mg/dL) at 2 h in a 75-g oral glucose tolerance test, or typical diabetes mellitus symptoms together with a casual plasma glucose level of >11.1 mmol/L (200 mg/dL).

Past and current alcohol intake was assessed at the time of the first visit and at each following visit. The assessment of alcohol consumption was made according to patient declarations. We defined alcohol drinkers as subjects who still consumed alcohol at least one time each week after surgery.

Gastrointestinal bleeding (GI bleeding) was defined as at least one upper gastrointestinal bleeding occurred before or after operation.

Futher more, tissue microarrays of 152 cirrhotic liver tissues were gifted from the West China Hospital with complete patients’ authorization and full ethical approval of the Institutional Clinical Ethics Review Board of Sichuan University. The detail clinical information has been described in a previous article^20^. In brief, from 1995 to 2009, 152 HBV-positive cirrhotic liver tissues with complete clinical and follow up data were included in these tissue microarrays. All patients were followed up until May 2014, with a median observation time of 84.5 months. The same follow-up protocol with Chongqing cohort was adopted. In total 21 patients were excluded in this study because they were not treated with Hassab procedure (9 liver transplantation, 3 simple splenectomy, 5 hepatectomy, 1 portacaval shunt, and 3 unclear operation information).

All human studies presented in our manuscript have been approved by the appropriate ethics committee and performed in accordance with the ethical standards established in the 1964 Declaration of Helsinki and its later amendments.

### Immunohistochemical staining

Immunohistochemical staining of tissue microarrays was carried out using a two-step protocol. Following antigen retrieval, the slices were incubated with primary antibody overnight at 4 °C. Negative controls were prepared by replacing the primary antibodies with PBS. To evaluate the expression of p-AMPK, tissue microarrays were assessed independently by two experienced pathologists with minimal inter-observer variability, and we used a semi-quantitative assessment method wherein the scoring parameters included staining intensity (range 0–3: 0—negative, 1—weak, 2—moderate, and 3—strong) and the percentage of positive cells (range 1–4: 0—negative or <5%, 1—6%–25%, 2—26%–50%, 3—51%–75%, and 4—76–100%). We added the percentage of positive cells to the intensity score to determine the final staining scores. Total score <4 was defined as low p-AMPK expression, while a score ≥4 was defined as high p-AMPK expression^21,22^. α-SMA^+^ activated hepatic stellate cells, collagen stain area, F4/80^+^ macrophages, and p62 positive cells were quantified using Image J software by applying the appropriate pixel threshold equally on all selected pictures and using measure function to calculate the covered area. Immunohistochemical staining quantification was performed on three randomly selected high-power fields per liver section. Data are represented as % of covered area for each cell type over total tissue area^23^.

### Animal model of hepatic carcinogenesis

Male C57BL/6 mice were obtained from Chongqing Medical University. To induce hepatic carcinogenesis, 4-week-old C57BL/6 male mice received 75 mg/kg DEN (Sigma-Aldrich, N0258) in two divided doses and CCL4 (mixed with olive oil in a 1:4 ratio, 0.08 ml/10 g) twice a week until 20 weeks. After 8 weeks, fresh liver tissues were obtained through liver biopsy. Immunohistochemical staining was used to detect the level of AMPK phosphorylation. The same criteria with human tissue microarrays were adopted to distinguish the level of p-AMPK expression. In mice with p-AMPK high expression, dorsomorphin (Abcam, ab120843, 0.2 mg/kg d) was used to inhibit p-AMPK, or PBS as a control. In mice with p-AMPK low expression, metformin (Sigma, PHR1084, 250 mg/kg d) or AICAR (Sigma, A9978, 2.5 mg/kg d) were used to activate AMPK. For autophagy inhibition, mice received 50 mg/kg of chloroquine (Santa Cruz Biotechnology, sc-205629) via intraperitoneal injection once every 3 days along with a concurrent application of metformin. All mice were killed at 32 weeks. All animal experiments complied with the ARRIVE guidelines and was carried out in accordance with the U.K. Animals (Scientific Procedures) Act, 1986 and associated guidelines.

### Western blot analysis

Liver tissues were used for harvesting total protein extraction and estimation. Polyacrylamide gel electrophoresis (PAGE) was performed, and PAGE gels were transferred to nitrocellulose membranes. Following transfer, membranes were blocked with 5% nonfat milk, incubated with antibodies for AMPK (CST, 2532), p-AMPK (CST, 2535), ACC (CST, 3662), mTOR(CST, 2983), p-mTOR(CST, 5536), RAGE(CST, 6996), CD44(ab157107), DLK1(CST, 2069), LC3B (CST, 2775), p62 (CST, 5114), and antibody for GAPDH (CST, 5174) was used as a loading control. The PVDF membranes were visualized using the Chemico-EQ system (Bio-Rad, USA). The targeted protein bands were analyzed and quantified using Image Lab 3 software (Bio-Rad, the USA).

### Serum analysis

The levels of alanine aminotransferase (ALT), total bilirubin (TBIL), albumin (ALB), and triglycerides were detected by an automatic biochemical analyzer (Beckman, USA).

### TEM analysis

The liver tissues were fixed with 2.5% glutaraldehyde Ultrathin sections were cut and doubly stained with uranyl acetate and lead citrate. For autophagic vacuole quantification, 10 micrographs, primary magnification ×2000, were randomly taken from each sample and the total amount of autophagic vacuoles was counted.

### Statistics analysis

The primary outcome was HCC occurrence after Hassab procedure. The significance of intergroup differences in continuous data were assessed using the Student's *t* test or Mann–Whitney U test, while that of differences in categorical data were assessed using the chi-squared test or Fisher’s exact test (two-tailed). Survival analysis was performed using the Fine and Gray model by accounting for the competing risk of death^24^.

Continuous variables are described as mean±standard deviation, and categorical variables are expressed as frequency (%). The results of survival analysis are described as hazard ratio (HR) and 95% confidence interval (CI). The multivariable model was generated by using the backward selection method. All reported *P* values were two-sided and *P* < 0.05 was considered significant. Statistical analyses were performed using SPSS 22.0 or R 3.4.0 (http://www.R-project.org/).

## Electronic supplementary material

Supplementary Information
