# Supplementary material for: Predictive and preventive significance of AMPK activation on hepatocarcinogenesis in patients with liver cirrhosis
Source: Cell Death Dis. 2018 Feb 15;9(3):264. doi: 10.1038/s41419-018-0308-4 (PMC5833839; doi:10.1038/s41419-018-0308-4)
Supplement: Supplementary file 1 — Supplementary Information [file 41419_2018_308_MOESM1_ESM.pdf]

Supplementary Information

Predictive and preventive significance of AMPK activation on hepatocarcinogenesis in patients with liver cirrhosis

Xiaoli Yang, Yan Liu, Menghui Li, Hao Wu, Yunbing Wang, Yu You, Peizhi Li, Xiong Ding, Chang'an Liu, Jianping Gong

Supplementary Figures & Legends

Supplementary Figure 1

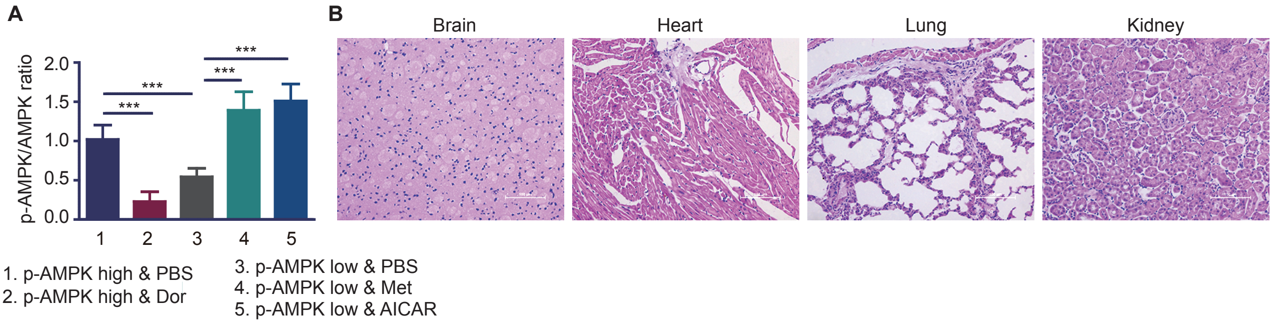

Supplementary Figure 1. (A). Quantitation of AMPK phosphorylation (mean ± SD, n= 6-9 mice per group). (B). Representative images of brain, heart, lung and kidney section from mice that were stained with H.E. ***P<0.001.

Supplementary Figure 2

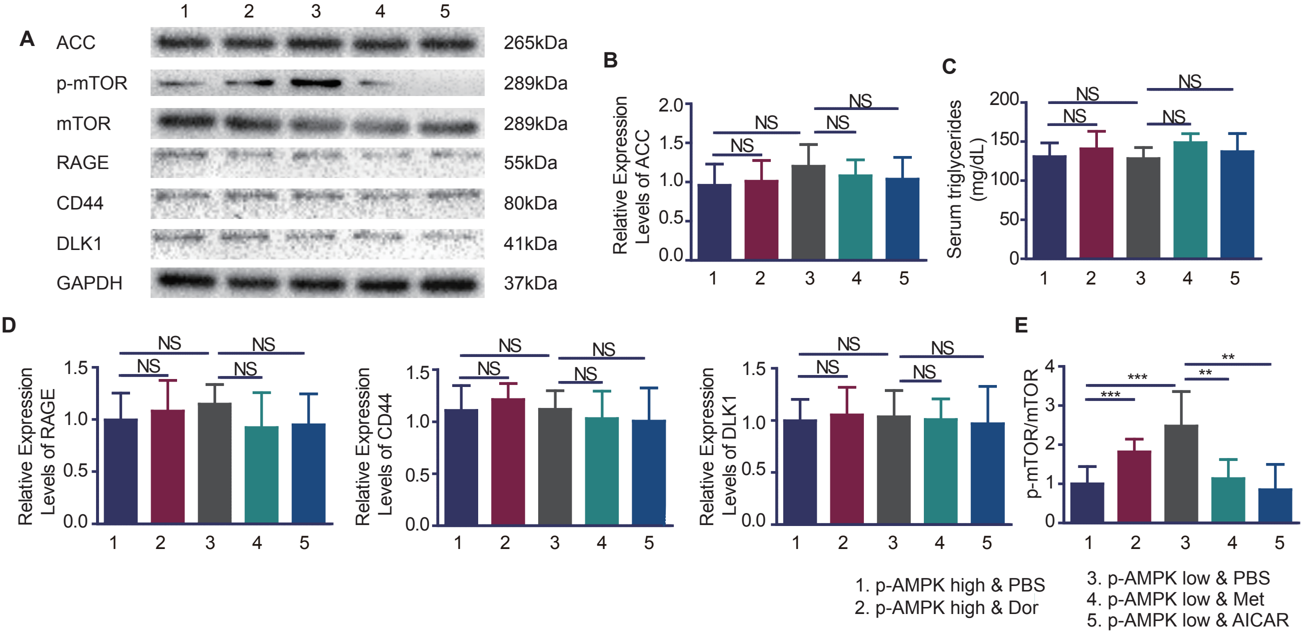

Supplementary Figure 2. (A). Liver lysates from mice were analyzed by immunoblot with the indicated antibodies. GAPDH was used as loading control. (B). Quantitation of ACC expression (mean ± SD, n= 6-9 mice per group). (C). Serum levels of triglycerides were measured (mean ± SD, n= 6-9 mice per group). (D-E). Quantitation of indicated protein expression and mTOR phosphorylation. (mean ± SD, n= 6-9 mice per group). ***P<0.001, **P<0.01, NS P>0.05.

Supplementary Figure 3.

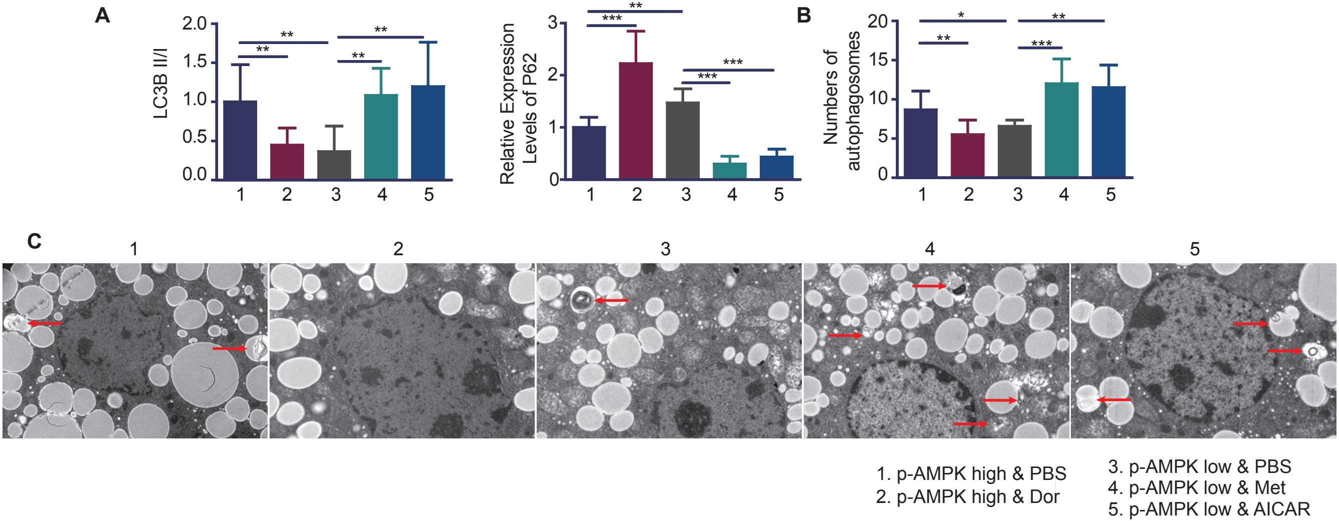

Supplementary Figure 3. (A). Quantitation of LC3B II/I ratios P62expression (mean ± SD, n= 6-9 mice per group). (B-C). Representative transmission electron micrographs showing autophagosomes. Autophagosomes are indicated by arrows. The numbers of autophagosomes were determined (mean ± SD, n= 6-9 mice per group). ***P<0.001, **P<0.01, *P≤0.05.

Supplementary Figure 4

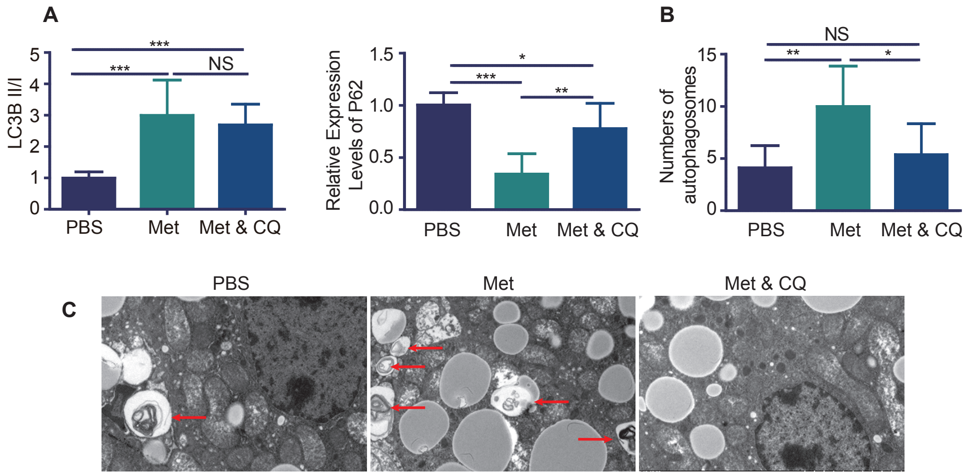

Supplementary Figure 4. (A) Quantitation of LC3B II/I ratios P62expression (mean ± SD, n =8 mice per group). (B-C). Representative transmission electron micrographs showing autophagosomes. Autophagosomes are indicated by arrows. The numbers of autophagosomes were determined (mean ± SD, n =8 mice per group). ***P<0.001, **P<0.01, *P≤0.05, NS P>0.05.
